# Machine Learning Approach for Differentiation of Pheochromocytoma from Adrenocortical Cancer and Non-Functioning Adrenal Adenomas

**DOI:** 10.3390/life16010164

**Published:** 2026-01-19

**Authors:** Timur Nurkhabinov, Irena Ilovayskaya, Anna Lugovskaya, Victor Popov, Lidia Nefedova

**Affiliations:** 1 Faculty of Mechanics and Mathematics, M.V. Lomonosov Moscow State University, 119991 Moscow, Russia; nurkhabinov.tt18@physics.msu.ru; 2 Department of Neuroendocrine Diseases, M.F. Vladimirsky Moscow Regional Research Clinical Institute, 129110 Moscow, Russia; doc@irenailov.ru (I.I.); annettae.leto@gmail.com (A.L.); 3 Faculty of Physics, M.V. Lomonosov Moscow State University, 119991 Moscow, Russia; masterlu@mail.ru; 4 Faculty of Biology, M.V. Lomonosov Moscow State University, 119991 Moscow, Russia

**Keywords:** pheochromocytoma, adrenocortical carcinoma, non-functioning adrenal adenoma, prognostic factors, machine learning

## Abstract

Background: The differentiation of pheochromocytoma (PCC) from other adrenal lesions, particularly in incidentalomas with non-benign radiological characteristics (size > 4 cm or density > 10 HU), remains a clinical challenge. The study aimed to develop and validate an interpretable machine learning (ML) model for pairwise differentiation of PCC from adrenocortical carcinomas (ACCs) and non-functioning adrenal adenomas (NAAs) and to identify the most important clinical features. Methods: We analyzed a dataset of 50 clinical, laboratory, and radiological parameters from 123 patients with histologically verified adrenal tumors (63 PCC, 30 ACC, 30 NAA). Four classifiers—Logistic Regression (LR), Random Forest (RF), Linear Discriminant Analysis (LDA), and Extreme Gradient Boosting (XGBoost)—were trained for binary classification tasks (PCC vs. ACC, PCC vs. NAA, ACC vs. NAA) using a robust nested stratified cross-validation pipeline to ensure generalizability and avoid overfitting. Results: All four models showed strong predictive performance, with discrimination (AUC) more than 0.8. Our analysis, based on the interpretable LR model, identified the key discriminators differentiated PCC from both ACC and NAA: maximum systolic blood pressure, grade 3 hypertension, headache, palpitation, tachycardia, male sex, and concomitant gastric and duodenal ulcers. In contrast, lower back pain and general weakness were strong signs of lower probability of PCC. The tumor density specifically differentiated PCC from NAA, whereas tumor size was an important marker for distinguishing PCC and ACC. Conclusions: We developed robust ML models capable of accurately differentiating PCC from other adrenal tumors in complex cases. The models provide a clinically actionable tool for pre-surgical decision support. Furthermore, the identification of key discriminative features enhances the clinical understanding of PCC and facilitates its differential diagnosis prior to histological verification.

## 1. Introduction

In recent years, the widespread use of computed tomography (CT) and magnetic resonance imaging has led to the enhanced detection of adrenal incidentalomas in the general population [1,2,3]. According to the results of a Minnesota population-based cohort study, adrenal tumor standardized incidence rates increased 10 times from 1995 to 2017 [4]. Another study from a large UK university teaching hospital demonstrated that scans performed increased 7.7% year-on-year from 2015 to 2019, with a more pronounced increase in the number with adrenal incidentalomas (14.7% per year) [5]. And discovered adrenal tumors require subsequent evaluation for hormonal activity and malignancy risk [1,2].

The European Society of Endocrinology clinical practice guidelines give a strong recommendation that noncontrast CT is consistent with a benign adrenal mass if the tumor size is <4 cm, has a homogenous appearance, and the CT native density is ≤10 Hounsfield units (HU) [1]. Adrenal tumors with a different CT appearances need additional consideration.

Benign non-functioning adrenal adenomas (NAAs) are the most common adrenal incidentalomas, with a reported frequency of up to 70–76% [6]. Among adrenal incidentalomas with non-benign CT characteristics, the most prevalent are pheochromocytomas (PCC), followed by adrenocortical carcinomas (ACC) [7].

PCC is a catecholamine-secreting neuroendocrine non-epithelial neuroendocrine neoplasm. The recent systematic review and meta-analysis of observational studies showed that the pooled prevalence of pheochromocytoma was 19.8 (95% CI: 9.6–40.8) cases per 1,000,000 individuals, and the incidence rate was 1.9 (95% CI: 1.2–2.6) cases per million person-years [8]. According to the updated 2022 WHO classification of neuroendocrine tumors and tumors of endocrine organs, all PCCs are considered as malignant and potentially metastatic tumors [9]. Therefore, early recognition of PCC among adrenal incidentalomas is crucial for determining the appropriate treatment strategy and monitoring to prevent tumor progression and metastasis. Furthermore, surgical intervention for PCC typically requires specific preoperative medical preparation because excessive catecholamine release during the procedure can lead to life-threatening cardiovascular complications [10].

Machine learning (ML) methods have become an increasingly important tool for enhancing the reliability of medical diagnosis. Various ML techniques are actively employed to differentiate PCC from lipid-poor adrenal adenomas and other adrenal tumors [11,12,13] and are further applied to stratify the risk of disease progression in cases of metastatic PCC [14,15]. Provided that sufficiently large datasets are available, ML can be leveraged to create tools that assist specialists in both medical research and clinical practice. Commonly used methods include algorithms such as Logistic Regression [11,12,13], Support Vector Machines [11,12,14], and Random Forests [11,12,15], as well as Neural Networks [16,17]. Having established themselves as reliable diagnostic aids, the predictive quality of these ML methods can be further improved with larger amounts of data.

This work presents a pairwise comparison of diagnoses for PCC, ACC, and NAA in patients with adrenal incidentalomas with non-benign CT appearance using acquired data and ML methods. The study aims to advance knowledge and improve the reliability of ML models in differentiating PCC from ACC and NAA, based on clinical, laboratory, and instrumental findings. The training data comprised parameters related to adrenal tumors, with the goal of identifying clinical (pre-testing) predictors of PCC in patients exhibiting an adrenal tumor larger than 40 mm and/or a noncontrast CT density greater than 10 HU.

We developed an ML pipeline to perform feature selection, tune hyperparameters, and assess the distribution of quality metrics on a limited dataset. The pipeline is also robust to outliers. As a pilot study, this work lays the foundation for developing a reliable model to assist in clinical diagnosis as more data becomes available.

## 2. Materials and Methods

*Patients*. The study cohort was derived from patients who underwent adrenalectomy at the M.F. Vladimirskiy Moscow Regional Research and Clinical Institute over a 12-year period (2011–2023; *n* = 265).

The inclusion criteria were as follows: a histologically and immunohistochemically verified diagnosis of PCC, ACC, and NAA; an adrenal tumor with an unenhanced CT density of >10 HU and/or size of >40 mm; and available data from clinical and biochemical examinations.

Exclusion criteria were other adrenal tumors (or metastases from other malignancies) confirmed by histology and immunohistochemistry; lack of histological or immunohistochemical verification of adrenal tumor; and absence of data on tumor density and size from CT scans.

The selection process was as follows: first, patients with adrenal tumors exceeding 40 mm in diameter and/or demonstrating a pre-contrast attenuation of >10 HU on computed tomography were identified (*n* = 156). Subsequently, the following exclusion criteria were applied: (1) histological and immunohistochemical confirmation of other adrenal tumors or metastases from non-adrenal malignancies (*n* = 23); (2) absence of definitive data on tumor size or density from CT imaging (*n* = 2); and (3) lack of essential clinical or biochemical information (*n* = 4). Consequently, the final study group consisted of 123 patients (88 women and 35 men, aged 22–81 years). Based on the histological/immunohistochemical verification, patients were divided into three groups: 63 with PCC (40 women, 23 men, aged 22–81 years), 30 with ACC (23 women, 7 men, aged 28–68 years), and 30 with NAA (26 women, 4 men, aged 27–79 years).

*Study design.* A single-center, retrospective, observational non-interventional cohort study.

*Clinical methods.* Our study assessed about 50 clinical and biochemical indicators. The following clinical manifestations were assessed: headache, palpitations, sweating, facial paleness and redness, nausea, vomiting, abdominal pain, hand numbness, body tremors, anxiety, panic attacks, muscle weakness, heat sensations, lower back pain, general weakness, chills, tinnitus, seeing spots, unexplained weight loss, dyspnea, chest pain, constipation, dizziness, systolic and diastolic blood pressure, heart rate, and the presence, type (continuous or paroxysmal), and grade of arterial hypertension. A broad panel of concomitant diseases was also evaluated, including diabetes mellitus, thyroid nodules, medullar thyroid carcinoma, primary hyperparathyroidism, concomitant non-functioning adrenal adenomas, pituitary tumors, neuroendocrine tumors, chronic heart failure, cerebrovascular accident, cholelithiasis, gastroduodenal ulcers, urolithiasis, chronic pyelonephritis, respiratory diseases, other oncological diseases, autoimmune disorders, and chronic infectious diseases.

Daily urinary excretion of fractionated metanephrines and normetanephrines was measured using high-performance liquid chromatography–tandem mass spectrometry (HPLC-MS/MS). The reference values were <320 μg/day for metanephrines and <390 μg/day for normetanephrines. CT was performed using Aquilion Prime Canon (Toshiba, Japan), 160-slice, with a contrasting agent Omnipaque 350 (GE HealthCare, Norway) according to the standard protocol. We collected data on the size, native (non-enhanced) density in HU, and contrast of accumulation of contrast agent by the adrenal lesions The volume of adrenal tumors was calculated using the formula: volume (cm^3^) = (length, mm) × (width, mm) × (thickness, mm) × 0.0005.

*Statistical Analysis and Predictive Modeling.* To identify features that most effectively help distinguish diagnoses in the data we collected, statistical analyses were conducted using R language (version 4.3.3) in RStudio 2024.04.2 (version 2024.04.2+ 764). We used non-parametric statistical tests for our data analysis, and results are presented as the median [interquartile range (IQR) 25–75%]. To determine whether there are statistically significant differences, quantitative variables were compared using the Kruskal–Wallis test for multiple comparison, followed by post hoc pairwise Dunn tests with Holm–Bonferroni correction to control the error rate. Qualitative features were compared using the Chi-square test or Fisher’s exact test, as appropriate. A *p*-value < 0.05 was considered statistically significant to ensure that the identified differences are not due to chance. Predictive modeling was performed using Python (version 3.9.12). The criteria for selecting classifiers for this work were the ability to obtain feature importance for the model and the widespread use of these methods in similar studies. We trained four classification models: Logistic Regression (LR), Random Forest (RF) Classifier, Linear Discriminant Analysis (LDA), and Extreme Gradient Boosting (XGboost) Classifier, for each pair of three diagnostic groups (PCC vs. ACC, PCC vs. NAA, ACC vs. NAA). To address moderate class imbalance, class weights were balanced during model training. A stratified nested cross-validation pipeline was implemented to ensure robust performance estimation and prevent overfitting. The pipeline included data preprocessing, hyperparameters optimization, and model validation. Dataset description (feature coding and percentage of missing data) is presented in Table S1. Missing data were imputed using the K-nearest neighbor (KNN) algorithm, and features were standardized to facilitate model convergence and enable the interpretation of LR coefficients.

Although the number of neighbors for KNN should be selected via validation for each specific dataset to improve metric quality, our pilot work did not aim to create the most accurate models (especially given the data volume). Instead, we focused on comparing the performance of the considered models on equally filled data and calculating feature importance in classifying different diagnosis pairs. Therefore, the number of neighbors was set to 3 as a common initial approximation.

**Figure 1:**
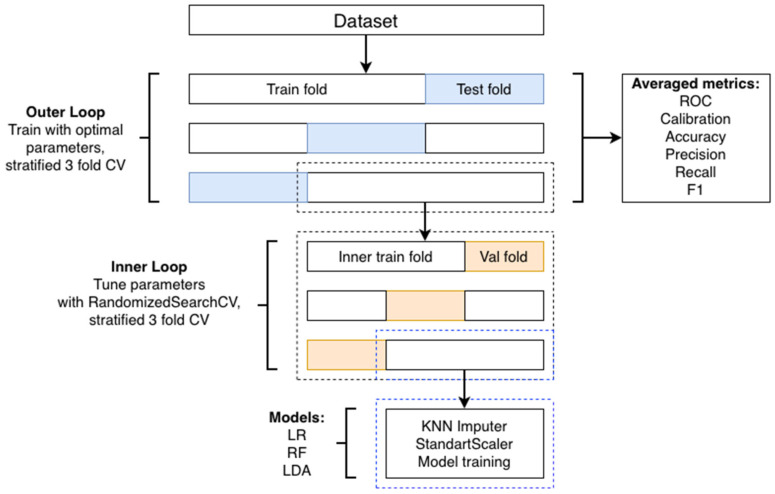
Stratified nested cross-validation flowchart.

*Model Validation and Hyperparameters Optimization.* Stratified nested cross-validation was employed to provide an unbiased estimate of the models’ performance and prevent data leakage from the hyperparameter tuning process. A flowchart demonstrating a pipeline for data processing, model training, and validation is shown in Figure 1.

The outer loop used stratified 3-fold cross-validation to assess generalization performance. For each outer fold of nested cross-validation, the training subset underwent hyperparameter tuning via the inner loop. Hyperparameters were sampled randomly from their specified distributions during the tuning process. Table 1 presents the hyperparameter search spaces for the respective models, as well as the optimal sets of these parameters found in the inner loops. As optimal sets, the ones selected were those in which the total number of repetitions in the other sets found in the inner loops was maximum. Hyperparameter optimization was performed via randomized search from the Scikit-learn library [18] (RandomizedSearchCV) with ten iterations for LR, RF, and LDA and twenty iterations for XGBoost within an inner stratified K-fold cross-validation scheme comprising three folds. This approach minimizes overfitting and provides a reliable estimate of optimal hyperparameters, while reducing computational cost compared to exhaustive grid search. Outer loop was performed via 3-fold stratified cross-validation to evaluate the generalization performance of the tuned model, avoiding optimistic bias. Data leakage was mitigated as follows: the outer cross-validation loop divided the entire dataset into training and testing sets. The test data was only used for final evaluation and was not involved in model training or hyperparameter tuning. The inner cross-validation operated only on the training set of the current outer fold. This ensures that hyperparameter tuning occurs strictly within the training data. All preprocessing steps—such as imputation and feature scaling—fit only on the training data of each fold and then applied to validation data, preventing information leakage from the test set into the training process.

For the RF Classifier (RandomForestClassifier [18]), the following hyperparameters were tuned: the number of trees (n_estimators), the maximum tree depth (max_depth), the number of features considered at each split (max_features), the minimum samples required to split an internal node (min_samples_split), and the minimum samples required at a leaf node (min_samples_leaf). The class_weight parameter was set to ‘balanced’. For LR (LogisticRegression [18]), the following were tuned: the inverse of regularization strength (classifier__C), which controls the amount of regularization, and the type of regularization penalty (classifier__penalty), with options being ‘l1’ (Lasso) or ‘l2’ (Ridge). Tuning the penalty type enables implicit feature selection through sparsity (L1) and handles multicollinearity (L2). We also tuned the optimization algorithm (classifier__solver) and the maximum number of iterations for convergence (classifier__max_iter). The class_weight parameter was set to ‘balanced’. For LDA (LinearDiscriminantAnalysis [18]), the following were tuned: the solver for computing discriminant functions (classifier__solver) and the regularization parameter (classifier__shrinkage) to improve model stability, particularly with small datasets or in the presence of multicollinearity. For each algorithm, the optimal hyperparameters found were used to train a model on the entire training fold. This model was then evaluated on the corresponding test fold. This process was repeated for all outer folds, and the performance metrics were aggregated by averaging to assess the overall generalization capability. For XGBoost (XGBClassifier [19]), the following hyperparameters were tuned: the number of trees in the model (classifier__n_estimators); the maximum depth of each tree, which limits tree growth to prevent overfitting (classifier__max_depth); the step size for updating the model (classifier__learning_rate), which controls training speed and balance between bias and variance; the fraction of training samples used for building each tree (classifier__subsample), which helps reduce overfitting; the fraction of features sampled for each tree (classifier__colsample_bytree) to increase model diversity; the minimum sum of instance weights needed in a leaf (classifier__min_child_weight); the minimum loss reduction required to make a split (classifier__gamma); classifier__reg_alpha: the L1 regularization term, which promotes sparsity in the model; and classifier__reg_lambda: the L2 regularization term, which helps prevent overfitting by shrinking weights.

***Performance Metrics***. Classifier performance was evaluated using multiple metrics to ensure a comprehensive assessment. The following metrics were calculated: accuracy, precision, recall, F1-score, and the Brier score for calibration. The formulas for these metrics are provided in reference [20].

$$Accuracy=\;\frac{tp+tn}{tp+\;fn+fp+tn\;}$$

$$Precision=\;\frac{tp}{tp+\;fp\;}$$

$$Recall=\;\frac{tp}{tp+\;fn\;}$$

$$F1score\;=\;2\frac{Precision\cdot Recall}{Precision+Recall}$$

$$Brier\;score\;=\;\frac{1}{N}\sum_{i=1}^{N}\sum_{c=1}^{C}{\left(y_{ic}-p_{ic}\right)}^{2}$$

tp—true positive;

tn—true negative;

fp—false positive;

fn—false negative;

*N*—number of dataset samples;

$y_{ic},\;p_{ic}$—target value and model-estimated probability of belonging to a given class *c*.

For probabilistic calibration, we implemented the Brier score as a custom scorer. Calibration visualization: For each outer fold, predicted probabilities for the positive class are binned into 10 intervals. For each bin, we calculated the mean predicted probability and the true fraction of positive samples in that bin. Later, the calibration information from all folds was combined by interpolating these points onto common bins.

The area under the Receiver Operating Characteristic (ROC) curve was measured to quantify model discrimination. ROC curve visualization: For each fold of the outer cross-validation, the values of False Positive Rate (FPR) and True Positive Rate (TPR) are calculated, which form the ROC curve. However, the points of FPR can differ for each fold (due to different prediction distributions). To average the ROC curves across folds, an interpolation of the TPR is performed at evenly spaced values of FPR.

To understand the real-world clinical impact and the types of mistakes that models make confusion matrices were calculated for all models for each pair of the diagnoses. True positives, true negatives, false positives, and false negatives were averaged across all outer folds. Evaluating both discrimination and calibration, as well as other multiple performance metrics, gives a fuller picture of model quality.

## 3. Results

### 3.1. Clinical Characteristics

Patients’ age at diagnosis varied significantly across the groups (*p* = 0.015) and was 51 years (IQR 43.5–59) in the PCC group, 50 years (IQR 44–61.5) in the ACC group, and 59.5 years (IQR 50.5–70.8) in the NAA group, with the NAA group being significantly older than PCC (*p* = 0.014 after correction). A trend was observed towards a higher frequency of male sex in the PCC group (23/63, 36.5%) compared to the ACC (7/30, 23.3%) and NAA (4/30, 13.3%) groups (*p* = 0.054). Among the clinical characteristics and symptoms assessed, the prevalence of headache, palpitation, weakness, and lower back pain differed significantly between groups (Table 2). Headache and palpitation were significantly more common in the PCC group compared to the ACC and NAA groups (*p* < 0.001 and *p* = 0.016, respectively). In contrast, general weakness and lower back pain were significantly less associated with PCC compared to ACC and NAA (both *p* < 0.001). The prevalence of other examined symptoms was similar in the groups.

We found the high prevalence of arterial hypertension across all patient groups: PCC 92.0% (58/63), ACC 76.7% (23/30), and NAA 80% (24/30), with no statistically significant differences between groups (*p* = 0.093). Paroxysmal hypertension was the predominant type in all groups, and its prevalence was significantly different: 86.2% (50/58) in the PCC group, 78.2% (18/23) in the ACC group, and 58.3% (14/24) in the NAA group (*p* = 0.012). Both maximal systolic and diastolic blood pressure values were significantly higher in the PCC group compared to the ACC (*p* < 0.001 after correction) and NAA groups (*p* < 0.001 after correction) (Table 3). The severity of hypertension also differed, and the prevalence of grade 3 hypertension was significantly higher in the PCC group: 88.0% (51/58) vs. 56.5% (13/23) in the ACC group and 41.6% (10/24) in the NAA group (both *p* < 0.001). While the median heart rate was not distinct between the groups, the prevalence of tachycardia (heart rate > 90 bpm) was significantly higher in the PCC group (23.8%, 15/63) than in the ACC (10%, 3/30) and NAA (3%, 1/30) groups (*p* = 0.016).

Laboratory examination revealed catecholamines hypersecretion in 95.2% (60/63) of PCC cases, 3.3% (1/30) of ACC cases, and 14.0% (4/30) of NAA cases (see Table 3). A mixed biochemical type of PCC (hypersecretion of both metanephrines and normetanephrines) was the most common finding in PCC, observed in 56.7% (34/60) cases. Isolated normetanephrine elevation was found in 30% (18/60) of PCC cases, while isolated metanephrines elevation was present in 13.3% (8/60) of the cases. In the ACC group, one case exhibited elevated levels of both metanephrines and normetanephrines. In the NAA group, two patients had isolated normetanephrine elevation, and two others showed a mixed type of hypersecretion.

According to the CT results, there were statistically significant differences in maximum tumor size, volume, and unenhanced density among the groups (*p* < 0.001). Tumor size and volume were significantly lower in the PCC group compared to the ACC group (*p* < 0.001 after correction), but comparable to those in the NAA group (*p* = 0.953). In contrast, unenhanced CT density was similar between the PCC and ACC groups (*p* = 0.128 after correction), but was significantly higher in the PCC group than in the NAA group (*p* < 0.001 after correction).

Concomitant diseases occurred with similar frequency, except for gastroduodenal ulcers: its prevalence differed among the groups (*p* = 0.029), with a higher frequency in the PCC group compared to the ACC and NAA groups (both *p* = 0.047) (Table 4).

Thus, we obtained data on clinical, biochemical, and morphological characteristics, which were then included in the development of the ML models.

### 3.2. Model Performance

The ML models for this study were selected based on the research objectives. Logistic Regression (LR) was chosen for its several advantages in statistical modeling. A primary strength is its interpretability; the model coefficients provide direct insight into the relationship between input features and the predicted outcome probability. Additionally, LR is computationally efficient. The next method was Random Forest (RF) Classifier, a robust and widely adopted method for binary classification. By constructing an ensemble of decision trees and aggregating their predictions, it significantly reduces overfitting compared to individual trees and achieves high predictive accuracy. It is also resilient to noisy data and outliers. Linear Discriminant Analysis (LDA) was also employed. This technique is widely used for binary classification due to its simplicity, interpretability, and solid theoretical foundation. A key advantage lies in its ability to find a linear combination of features that maximally separates the two classes. While computationally efficient, LDA has certain limitations, including sensitivity to outliers, assumptions about feature distributions, and potential issues with small sample sizes. In this work, LDA was included for comparison with other models, and was also utilized for patients’ data visualization. The last model evaluated in this study was Extreme Gradient Boosting (XGBoost), a scalable tree-boosting system renowned for its superior performance on structured data. Key advantages include built-in regularization to mitigate overfitting, native handling of missing values, and class imbalance.

**Figure 2:**
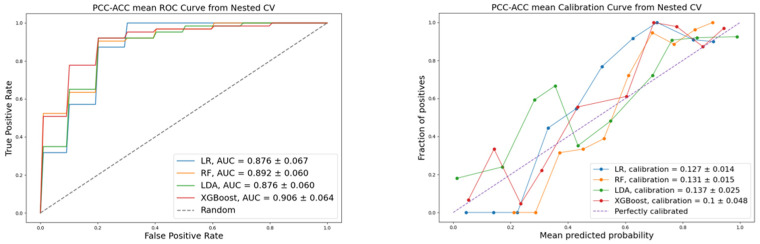
ROC and calibration: PCC vs. ACC classification.

**Figure 3:**
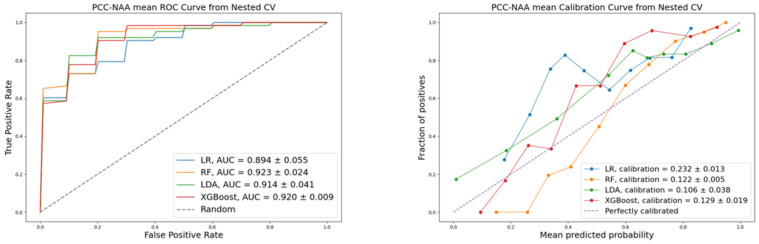
ROC and calibration: PCC vs. NAA classification.

**Figure 4:**
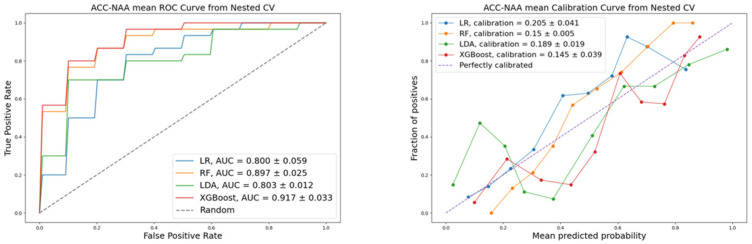
ROC and calibration: ACC vs. NAA classification.

To compare the performance of ML algorithms used in this work, we calculated the following quality metrics: accuracy, precision, recall, F1-score, calibration, and discrimination. Stratified cross-validation was applied to improve the objectivity of the classification quality assessment. Visualization of the ROC and calibration for the evaluated models are presented in Figure 2, Figure 3 and Figure 4, corresponding to each pair of the three diagnoses: PCC, ACC, and NAA.

The models’ metrics are presented in Table 5. The performance of the four machine learning models varied across the different classification tasks. For the PCC vs. ACC and ACC vs. NAA classifications, XGBoost demonstrated the highest performance across all metrics, indicating its superiority for this pairwise comparisons.

For the PCC vs. NAA classification, the LDA model showed the best accuracy (0.8817 ± 0.0402), precision (0.8836 ± 0.0383), recall (0.8817 ± 0.0402), F1-score (0.8823 ± 0.0396), and Brier score (0.1060 ± 0.0380). The RF model, however, achieved the highest discriminative power with an AUC of 0.9230 ± 0.0243.

From the obtained confusion matrices (Table 6), for the PCC vs. ACC and PCC vs. NAA classifications, RF on average makes fewer errors than other models in cases of actual positive diagnosis. At the same time, XGBoost made the fewest errors of this type for the ACC vs. NAA pair. Even though no single model universally dominated all tasks in other metrics, RF exhibits the fewest false negative errors for PCC, which is clinically crucial.

**Figure 5:**
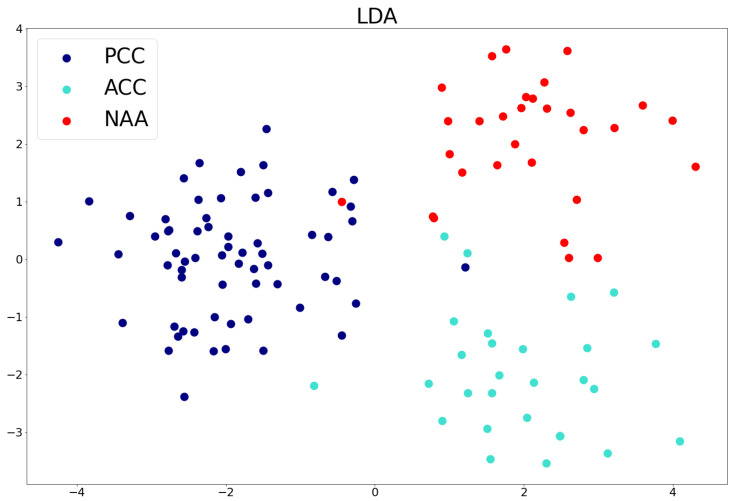
Linear Discriminant Analysis for PCC, ACC, and NAA.

The dimensionality reduction technique LDA was applied to visualize patient data clustering based on ground truth labels (Figure 5). Due to dimensionality reduction, the four ACC patients clustered into very close points, which caused the visualization to appear as if there were only 27 ACC patients on the graph.

One of the key objectives was to identify clinical features that can be used for differentiating PCC from ACC and NAA. To rank the features of the standardized data by their importance for classification, we used the Logistic Regression coefficients due to the model’s interpretability. We used stratified nested cross-validation with 10 outer folds and 5 inner folds for a more accurate estimation of the expected value of the coefficients. The feature importance values were averaged across the outer cross-validation folds.

**Figure 6:**
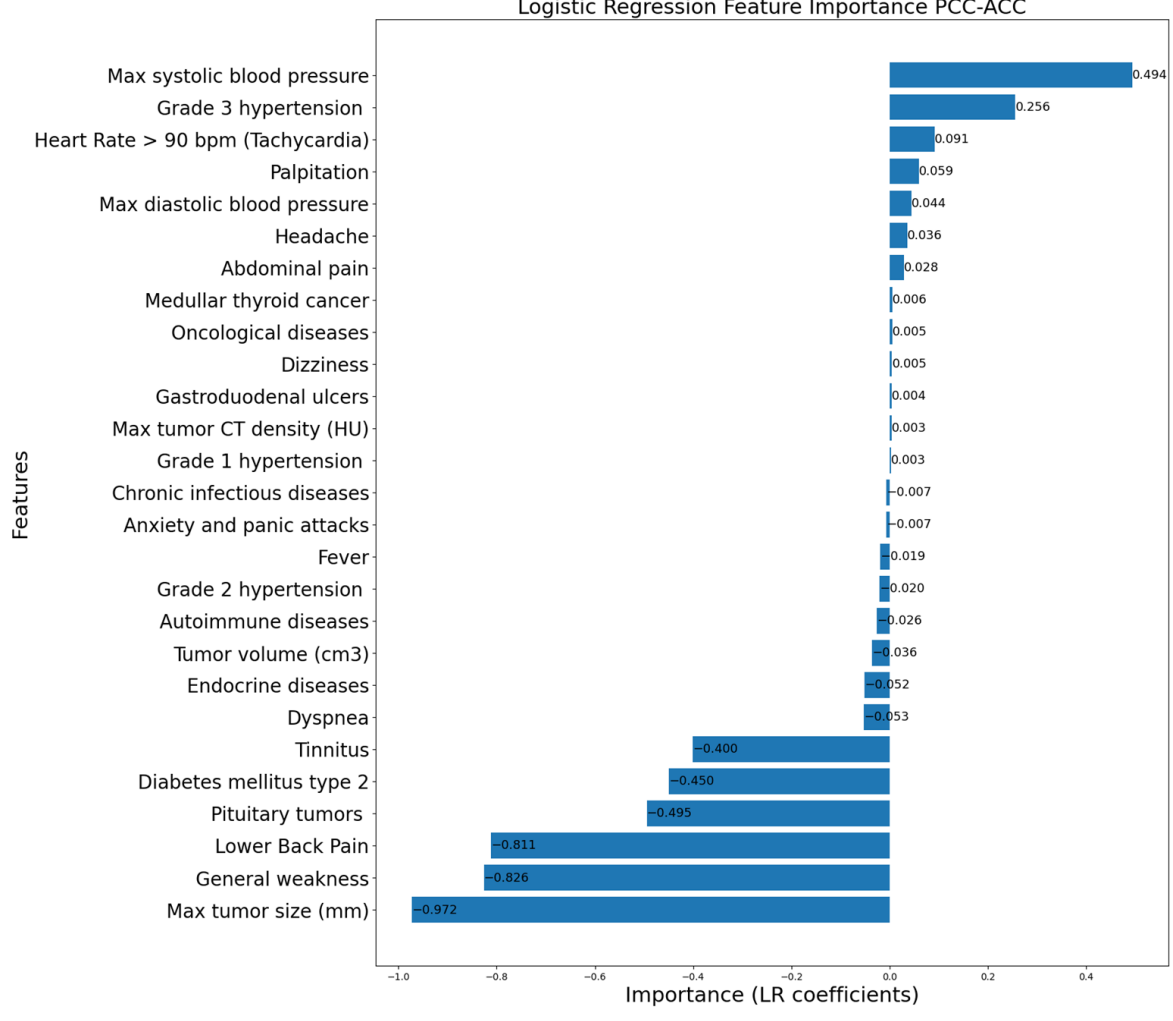
Logistic Regression feature importance for PCC vs. ACC.

**Figure 7:**
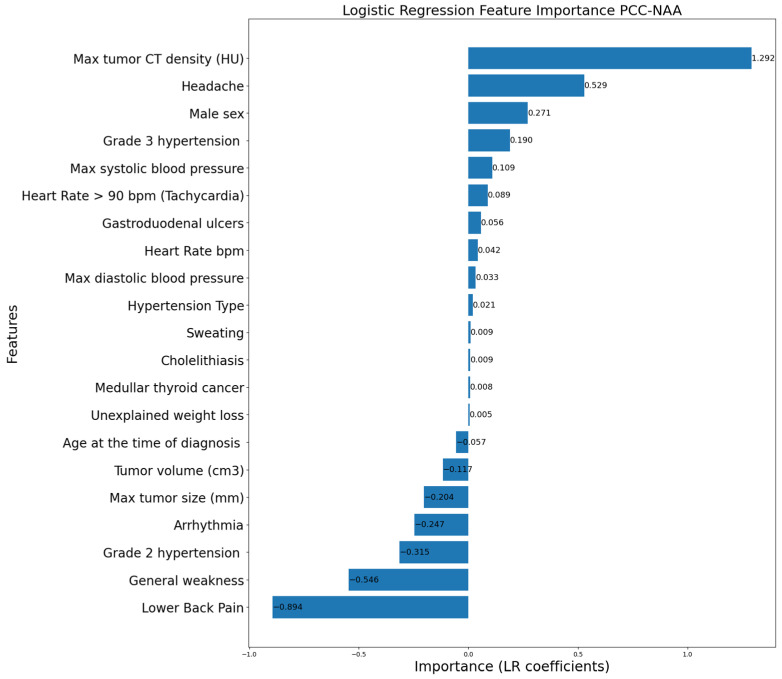
Logistic Regression feature importance for PCC vs. NAA.

**Figure 8:**
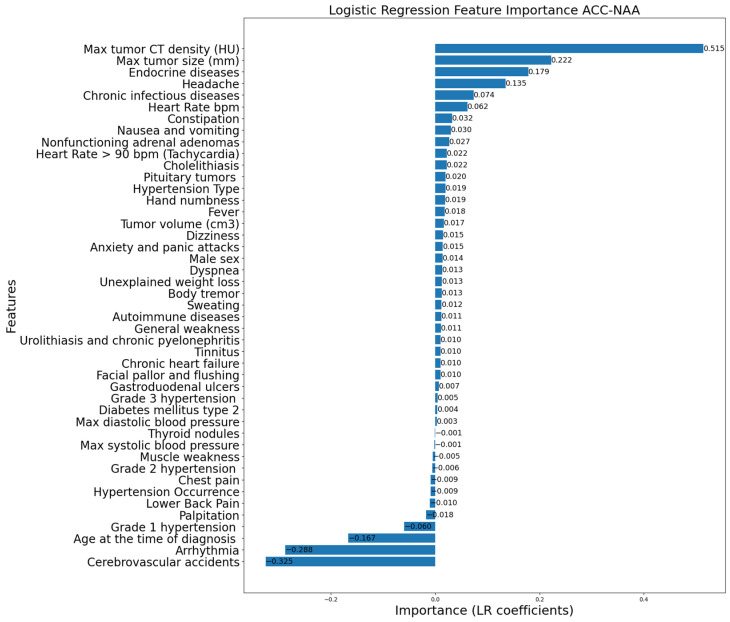
Logistic Regression feature importance for ACC vs. NAA.

Figure 6, Figure 7 and Figure 8 display the LR coefficients (only those with absolute values ≥ 0.001 are shown for better readability) for the features used for pairwise differentiation of PCC, ACC, and NAA. The absolute value of the coefficient reflects the feature’s contribution of in the separation. A positive coefficient value indicates a higher likelihood of the first diagnosis in a compared pair. On the contrary, a negative coefficient value indicates a preference for the second diagnosis in a compared pair.

According to the statistical analysis of clinical data (Table 2, Table 3 and Table 4), significant features distinguishing PCC from ACC and NAA were headache, lower back pain, palpitations, general weakness, maximum blood pressure, adrenal tumor volume and size, and tumor CT density. The LR coefficients were largely consistent with the statistical analysis, while highlighting key distinctions for each paired diagnosis. Overall, the LR model confirmed the importance of specific characteristics in differentiating these adrenal lesions.

## 4. Discussion

In our study, we compared patients with verified PCC diagnosis with patients with ACC (representing malignant adrenal lesions) and NAA (representing benign adrenal lesions). One aim of the current study was to validate ML models for pairwise differentiation of incidentally adrenal lesions with non-benign CT characteristics. Another key objective was to identify clinical features that could be relevant for recognizing PCC among adrenal incidentalomas.

The problem was framed as a pairwise classification task to distinguish PCC from ACC and NAA and to identify features enabling this differentiation. Even though XGBoost was most effective for distinguishing PCC from ACC and ACC from NAA in terms of accuracy, precision, recall, F1-score, ROC AUC, and Brier score, RF reducing false negative errors in PCC patient classification decreases the risk of missing truly ill PCC patients. LR provided the ability to extract feature importance from standardized data and made it possible to understand the sign of a feature’s contribution to the probability of a particular class, unlike, for example, RF feature importance. As a result, the model outcomes became clearer and more interpretable. These models could be further trained and applied in clinical practice. The identified markers could assist in better understanding the differences between PCC and other adrenal lesions, such as ACC and NAA. We combined traditional clinical and biochemical diagnostic methods with ML approaches to improve diagnostic performance.

Some recent publications that we have mentioned before demonstrated that ML models based on CT radiomics could differentiate lipid-poor adrenal adenomas from PCC. The study by Xiao et al. included 70 patients with lipid-poor adenomas and 60 PCC that was comparable the number of patients in our study, but the authors did not consider any clinical signs [11]. Liu et al. based their ML model on CT data from 188 lipid-poor adrenal adenomas and 92 PPC, but the authors mentioned an absence of clinical and biochemical data as a limitation of their study [12]. Altay et al. used ML to evaluate texture analysis for distinguishing various adrenal lesions including PCC and adrenal metastases from other tumors, but there were 19 cases of PCC only among 166 adrenal lesions included at the ML model [13]. So, we included clinical, anamnestic, biochemical, and radiological parameters of our 123 patients to develop an ML model for distinguishing PCC, ACC, and NAA among adrenal incidentalomas with non-benign characteristics according CT images.

The classical symptoms of PCC include hypertension, headaches, palpitations, and sweating. It is noteworthy that our model identified the importance of clinical PCC indicators: headaches and palpitation were important in all pairs, and the presence of lower back pain and general weakness strongly indicated against PCC. However, the landscape of other clinical signs was different. Although arterial hypertension is a primary clinical manifestation of PCC, it was equally prevalent across all examined groups. The ML algorithm did not consider hypertension *per se* or its paroxysmal type as specific indicators. High systolic and diastolic blood pressure, as well as grade 3 hypertension, appeared to be specific for PCC compared to ACC and NAA. Thus, the clinical indicator of PCC is the severity of arterial hypertension (grade 3), and not the presence or type of hypertension. (Figure 6 and Figure 7). The usual biochemical marker of PCC is a 2–3-fold increase in metanephrine and/or normetanephrine concentrations in blood plasma or daily urine. However, in our cohort of patients with verified PCC diagnosis, we observed 3/63 (4.8%) cases had both parameters within the reference range, so clinical markers of PCC are very important despite the ability of biochemical investigation.

Based on our data, male sex was a sign of higher probability of PCC compared to ACC and NAA. On multivariate analysis, male sex (among other factors) was found to be a statistically significant predictor of adrenal tumor malignancy [6]. However, ACC also showed a female predominance [21].

Our model assigned diagnostic significance to concomitant stomach and duodenum ulcers in PCC cases, which may raise clinicians’ awareness of this complication. Regarding the relationship between gastroduodenal ulcers and PCC, studies have shown that some PCC patients have elevated serum adrenaline and gastrin levels both basally and in response to food intake. These findings indicate that epinephrine stimulates gastrin secretion [22], providing a pathophysiological basis for the association between gastroduodenal ulcers and PCC. Chronic or paroxysmal catecholamine excess causes vasospasm of the gastric mucosal vessels, leading to impaired microcirculation, ischemia, and the consequent formation of “stress” or “ischemic” ulcers. Thus, gastroduodenal ulcers represent a rare but justified pathophysiological feature of PCC that is often overlooked in standard diagnostic algorithms. Our results highlight the importance of a comprehensive differential diagnosis and consideration of associated comorbidities.

According to CT results, PCC size and volume were similar to NAA but significantly smaller than ACC. In contrast, unenhanced CT density of PCC was comparable to ACC and higher than that of NAA. Our ML model showed clear separation of PCC from ACC and NAA based on different CT characteristics: high noncontrast CT density of the tumor was very important to distinguish PCC and NAA, whereas tumor size was notable marker to differentiate PCC and ACC in adrenal tumors with high noncontrast CT density.

A recently published study by Iwamoto et al. developed an ML-based clinical model that combined CT imaging and clinical parameters for adrenal tumor classification [23]. The aim of the study was to evaluate whether the tumors were hormone-producing. This retrospective study involved 162 patients with different adrenal lesions, including 55 cases of NAA and 23 cases of PCC, verified after surgical treatment. In the cited study, maximal tumor size was similar in PCC and NAA, but they did not include patients with ACC and did not comment on CT density of adrenal tumors. Some clinical parameters in that study were similar to our research (sex, systolic blood pressure, body mass index, catecholamine increase), but they included some additional hormonal test (aldosterone–renin ratio, cortisol–ACTH ratio) that we did not use. In contrast, we focused on another wide spectrum of clinical signs and concomitant disorders to evaluate their clinical significance in differentiating specific types of adrenal lesions on preanalytical stage.

It should be noted that our study has several limitations. The most notable is the relatively small sample size, particularly for the ACC group (*n* = 30). This is an inherent challenge in studying rare tumors like ACC, with an annual incidence of only 1–2 cases per million people per year [24]. However, as demonstrated in other medical ML studies [25,26], robust models can be developed even with limited data when using appropriate validation techniques, such as the nested cross-validation employed here. Nevertheless, small sample sizes pose significant challenges for nested cross-validation. Vabalas et al. [27] demonstrate that while nested CV mitigates bias better than standard k-fold CV—yielding near-unbiased accuracies close to 50% on noise data—even nested procedures on number of samples less than 200 suffer high variance due to limited resampling stability. Therefore, the small cohort size necessitates external validation on larger, multicenter datasets to confirm the generalizability of our findings.

We framed the problem as three separate binary classification tasks rather than a single multi-class model. This approach was chosen for two primary reasons: First, given the limited sample size, especially for ACC, building a multi-class model would have been statistically underpowered and prone to overfitting. Second, from a clinical perspective, the pairwise differentiation often reflects the immediate diagnostic dilemma faced by physicians when specific tumor characteristics are observed.

Another limitation of our study was sample bias, as we included only patients with histologically verified tumors after surgical treatment. Most NAA patients typically do not require surgical treatment due to absence of hormonal hypersecretion and benign CT features. This limitation, however, can also be regarded as a strength of the study, as all diagnoses were surgically confirmed. In our cohort, the primary indication for surgery in adrenal lesion cases without proven hormonal activity was non-benign CT characteristics of adrenal tumors suspected of malignancy. Adrenal tumors with non-benign CT characteristics represent the most challenging clinical scenarios that would benefit from the decision support. We suggest that our data will help to recognize the type of adrenal tumor with more confidence that could lead to better preoperative management in case of PCC and/or avoid an operation in case of NAA.

We also excluded patients with other types of adrenal lesions such as metastases of extra-adrenal cancer, because PCC, ACC, and NAA are the most prevalent adrenal tumors, according to the experience of many centers including ours [3,4,5,6,7]. It is important to consider that the model’s performance might not generalize to all patients with incidentalomas and need to be confirmed on independent, prospective cohorts from multicenter studies.

## 5. Conclusions

We developed and validated robust ML models for the differential diagnosis of PCC versus ACC and NAA in patients with adrenal tumors exhibiting non-benign CT characteristics. The key discriminators that differentiated PCC from both ACC and NAA were maximum systolic blood pressure, grade 3 hypertension, headache, palpitation, tachycardia, male sex, and concomitant gastric and duodenal ulcers. In contrast, lower back pain and general weakness were strong signs of lower probability of PCC. Furthermore, high tumor CT density specifically differentiated PCC from NAA, whereas tumor size was an important marker for distinguishing PCC and ACC. These findings highlight the potential of integrating clinical data into a diagnostic algorithm. The proposed models form a foundation for a future clinical decision support system (e.g., a script utilizing models validated in real clinical settings), which could aid endocrinologists and surgeons in risk stratification and preoperative planning for patients with challenging adrenal incidentalomas.

## Figures and Tables

**Table 1:** Hyperparameter search space and optimal values.

		PCC vs. ACC	PCC vs. NAA	ACC vs. NAA
Models	Hyperparameters: Values	Optimal Values		
LR	‘classifier__C’: np.logspace (−4, 4, 10)	0.36	2.78	21.54
	‘classifier__penalty’: [‘l1’, ‘l2’]	l1	l2	l2
	‘classifier__solver’: [‘liblinear’, ‘saga’]	saga	liblinear	saga
	‘classifier__max_iter’: [100, 200, 500]	200	200	100
RF	‘classifier__n_estimators’: [100, 300]	100	100	300
	‘classifier__max_depth’: [3, 5, None]	5	None	5
	‘classifier__max_features’: [‘sqrt’, ‘log2’]	log2	log2	log2
	‘classifier__min_samples_split’: [2, 5]	5	2	5
	‘classifier__min_samples_leaf’: [1, 2]	1	1	1
LDA	‘classifier__solver’: [‘svd’, ‘lsqr’, ‘eigen’]	lsqr	svd	svd
	‘classifier__shrinkage’: [None, ‘auto’] + list (np.linspace (0.0, 1.0, 11))	0.1	None	None
XGBoost	‘classifier__n_estimators’: [100, 300, 500]	100	100	300
	‘classifier__max_depth’: [3, 5, 7, 9]	9	9	7
	‘classifier__learning_rate’: [0.01, 0.05, 0.1, 0.2]	0.2	0.2	0.05
	‘classifier__subsample’: [0.7, 0.8, 0.9, 1.0]	0.9	0.9	0.9
	‘classifier__colsample_bytree’: [0.7, 0.8, 0.9, 1.0]	0.8	0.8	0.8
	‘classifier__min_child_weight’: [1, 3, 5, 7]	1	1	1
	‘classifier__gamma’: [0, 0.1, 0.2, 0.5]	0	0.5	0.1
	‘classifier__reg_alpha’: [0, 0.1, 1.0]	0.1	0.1	0
	‘classifier__reg_lambda’: [0, 0.1, 1.0]	1.0	1.0	0.1

**Table 2:** Clinical symptoms in examined patients.

Parameters	Patients *n* (%)		
	PCC *n* = 63	ACC *n* = 30	NAA *n* = 30
* Headache	36 (57.1)	9 (30.0)	3 (10.0)
* Lower back pain	12 (19.0)	18 (60.0)	22 (73.3)
* Palpitation	21 (33.3)	4 (13.3)	3 (10.0)
* General weakness	10 (15.9)	19 (63.3)	17 (56.7)
Abdominal pain	5 (7.9)	2 (6.7)	3 (10.0)
Anxiety and panic attacks	3 (4.8)	2 (6.7)	0
Body tremor	3 (4.8)	2 (6.7)	1 (3.3)
Chest pain	3 (4.8)	1 (3.3)	2 (6.7)
Chills	3 (4.8)	0	0
Constipation	1 (1.6)	1 (3.3)	0
Dizziness	6 (9.5)	1 (3.3)	0
Dyspnea	0	1 (3.3)	0
Fever	0	1 (3.3)	0
Muscle weakness	5 (7.9)	5 (16.7)	3 (10.0)
Nausea and vomiting	5 (7.9)	3 (10.0)	0
Hand numbness	2 (3.2)	2 (6.7)	0
Facial pallor and flushing	6 (9.5)	1 (3.3)	0
Sweating	13 (19.0)	6 (20.0)	3 (10.0)
Tinnitus	0	1 (3.3)	0
Unexplained weight loss	1 (1.6)	1 (3.3)	0

* *p* < 0.001.

**Table 3:** Clinical, laboratory, and instrumental characteristics of examined patients.

Characteristics M ± SD Me [25%; 75%] Min–Max	Measurements in Patient Groups		
	PCC *n* = 63	ACC *n* = 30	NAA *n* = 30
* Max systolic blood pressure, mm Hg	204 ± 40 200 [181; 228] 113–300	170 ± 38 168 [150; 195] 110–260	165 ± 32 170 [143; 190] 110–220
* Max diastolic blood pressure, mm Hg	110 ± 19 110 [100; 120] 70–160	97 ± 29 100 [83; 100] 60–220	92 ± 17 100 [73; 100] 60–130
Heart rate, bpm	78 ± 16 75 [66; 89] 43–120	77 ± 11 74 [69; 84] 59–98	70 ± 10 70 [62; 77] 50–92
* Adrenal tumor volume, cm^3^	140.4 ± 283.4 65.2 [25.4; 120.1] 3.3–2070	569 ± 561.5 348.2 [120.2; 890.9] 7.6–1819.5	237.7 ± 754.3 55.5 [32.4; 116] 8–4132.3
* Max tumor size, mm	48 ± 22 45 [34; 56] 16–138	80 ± 37 78 [53; 112] 5–153	52 ± 34 42 [37; 54] 21–197
* Max tumor CT density, HU	43 ± 19 41 [34; 50] 4–96	37 ± 10 36 [30; 40] 18–67	20 ± 19 19 [5; 29] 0–90
* Daily urinary excretion of fractionated metanephrines, mcg/day	2637 ± 4921 1063 [233; 3080] 7–29; 812	100 ± 82 100 [68; 118] 6–455	128 ± 107 110 [59; 155] 9–400
* Daily urinary excretion of fractionated normetanephrines, mcg/day	3193 ± 5826 1417 [695; 2876] 189–41; 273	154 ± 134 127 [69; 200] 9–615	244 ± 219 211 [126; 314] 14–1115

* *p* < 0.001.

**Table 4:** Prevalence of comorbidities in the examined patient groups.

Concomitant Diseases	Patients *n* (%)		
	PCC *n* = 63	ACC *n* = 30	NAA *n* = 30
* Gastroduodenal ulcers	16 (25.4)	2 (6.7)	2 (6.7)
Autoimmune diseases	1 (1.6)	2 (6.7)	1 (3.3)
Cholelithiasis	3 (4.8)	4 (13.3)	1 (3.3)
Chronic heart failure	2 (3.2)	1 (3.3)	0
Chronic infectious diseases	1 (1.6)	1 (3.3)	0
Diabetes mellitus type 2	12 (19.0)	9 (30.0)	7 (23.3)
Medullar thyroid cancer	1 (1.6)	0	0
Neuroendocrine tumors	2 (3.2)	0	0
Non-functioning adrenal adenomas	2 (3.2)	10	0
Oncological diseases	9 (14.3)	2 (6.7)	2 (6.7)
Pituitary tumors	0	1 (3.3)	0
Primary hyperparathyroidism	3 (4.8)	0	0
Respiratory diseases	2 (3.2)	2 (6.7)	1 (3.3)
Thyroid nodules	13 (20.6)	5 (16.7)	7 (23.3)
Urolithiasis and chronic pyelonephritis	5 (7.9)	4 (13.3)	3 (10.0)
Cerebrovascular accident	3 (4.8)	0	2 (6.7)

* *p* < 0.01.

**Table 5:** Models’ quality metrics.

Model Name	Classification Type	Accuracy	Precision	Recall	F1-Score	ROC AUC	Brier Score
LR	PCC vs. ACC	0.850 ± 0.066	0.872 ± 0.067	0.850 ± 0.066	0.851 ± 0.064	0.876 ± 0.067	0.127 ± 0.014
RF		0.839 ± 0.046	0.843 ± 0.055	0.839 ± 0.046	0.831 ± 0.050	0.892 ± 0.060	0.131 ± 0.015
LDA		0.828 ± 0.015	0.835 ± 0.023	0.828 ± 0.015	0.829 ± 0.016	0.876 ± 0.060	0.138 ± 0.025
XGBoost		0.871 ± 0.091	0.882 ± 0.087	0.871 ± 0.091	0.873 ± 0.087	0.906 ± 0.064	0.100 ± 0.048
LR	PCC vs. NAA	0.753 ± 0.110	0.809 ± 0.092	0.753 ± 0.110	0.760 ± 0.106	0.894 ± 0.055	0.232 ± 0.013
RF		0.860 ± 0.015	0.861 ± 0.014	0.860 ± 0.015	0.856 ± 0.019	0.923 ± 0.024	0.122 ± 0.005
LDA		0.882 ± 0.040	0.884 ± 0.038	0.882 ± 0.040	0.882 ± 0.040	0.914 ± 0.041	0.106 ± 0.038
XGBoost		0.817 ± 0.055	0.860 ± 0.018	0.817 ± 0.055	0.817 ± 0.052	0.920 ± 0.009	0.129 ± 0.019
LR	ACC vs. NAA	0.767 ± 0.024	0.773 ± 0.028	0.767 ± 0.024	0.766 ± 0.023	0.800 ± 0.059	0.205 ± 0.041
RF		0.800 ± 0.041	0.802 ± 0.041	0.800 ± 0.041	0.780 ± 0.041	0.897 ± 0.025	0.150 ± 0.005
LDA		0.783 ± 0.062	0.792 ± 0.061	0.783 ± 0.062	0.782 ± 0.063	0.803 ± 0.013	0.189 ± 0.019
XGBoost		0.817 ± 0.024	0.822 ± 0.023	0.817 ± 0.024	0.816 ± 0.024	0.917 ± 0.03	0.145 ± 0.039

**Table 6:** Confusion matrices.

	LR		RF		LDA		XGBoost	
	Predicted negative	Predicted positive	Predicted negative	Predicted positive	Predicted negative	Predicted positive	Predicted negative	Predicted positive
	PCC vs. ACC							
Actual negative	8.33 ± 1.25	1.67 ± 1.25	6.33 ± 1.25	3.67 ± 1.25	7.67 ± 0.94	2.33 ± 0.94	8.33 ± 1.25	1.67 ± 1.25
Actual positive	3.00 ± 2.16	18.00 ± 2.16	1.33 ± 0.94	19.67 ± 0.94	3.00 ± 0.82	18.00 ± 0.82	2.33 ± 2.05	18.67 ± 2.05
	PCC vs. NAA							
Actual negative	8.67 ± 1.25	1.33 ± 1.25	7.00 ± 0.82	3.00 ± 0.82	8.33 ± 0.47	1.67 ± 0.47	8.33 ± 1.70	1.67 ± 1.70
Actual positive	6.33 ± 2.36	14.67 ± 2.36	1.33 ± 0.47	19.67 ± 0.47	2.00 ± 0.82	19.00 ± 0.82	4.00 ± 2.94	17.00 ± 2.94
	ACC vs. NAA							
Actual negative	8.33 ± 0.47	1.67 ± 0.47	8.0 ± 0.0	2.0 ± 0.0	8.67 ± 0.47	1.33 ± 0.47	7.67 ± 0.47	2.33 ± 0.47
Actual positive	3.00 ± 0.00	7.00 ± 0.00	2.0 ± 0.82	8.0 ± 0.82	3.0 ± 0.82	7.0 ± 0.82	1.33 ±0.47	8.67 ± 0.47

## Data Availability

Additional information regarding the manuscript will be welcomed by the authors.

## Supplementary Materials

The following supporting information can be downloaded at https://www.mdpi.com/article/10.3390/life16010164/s1. Table S1: Dataset description.

## Abbreviations

The following abbreviations are used in this manuscript:

ACC	Adrenocortical carcinoma
AUC	Area under the curve
CT	Computed tomography
FPR	False Positive Rate
HU	Hounsfield Units
IQR	Interquartile range
KNN	K-Nearest Neighbors
LDA	Linear Discriminant Analysis
LR	Logistic Regression
ML	Machine Learning
NAA	Non-functioning adrenal adenoma
PCC	Pheochromocytoma
RF	Random Forest
ROC	Receiver Operating Characteristic
TPR	True Positive Rate
XGBoost	Extreme Gradient Boosting
